# Changing Epidemiology of Pulmonary Nontuberculous Mycobacteria Infections

**DOI:** 10.3201/eid1610.091201

**Published:** 2010-10

**Authors:** Rachel M. Thomson, on behalf of the NTM working group at the Queensland TB Control Centre, Queensland Mycobacterial Reference Laboratory

**Affiliations:** Author affiliation: Queensland Tuberculosis Control Centre, Brisbane, Queensland, Australia

**Keywords:** Atypical, epidemiology, bronchiectasis, pulmonary infection, tuberculosis and other mycobacteria, bacteria, CME, research

## MEDSCAPE CME

Medscape, LLC is pleased to provide online continuing medical education (CME) for this journal article, allowing clinicians the opportunity to earn CME credit. This activity has been planned and implemented in accordance with the Essential Areas and policies of the Accreditation Council for Continuing Medical Education through the joint sponsorship of Medscape, LLC and Emerging Infectious Diseases. Medscape, LLC is accredited by the ACCME to provide continuing medical education for physicians. Medscape, LLC designates this educational activity for a maximum of 0.5 *AMA PRA Category 1 Credits*™. Physicians should only claim credit commensurate with the extent of their participation in the activity. All other clinicians completing this activity will be issued a certificate of participation. To participate in this journal CME activity: (1) review the learning objectives and author disclosures; (2) study the education content; (3) take the post-test and/or complete the evaluation at www.medscapecme.com/journal/eid; (4) view/print certificate.

## Learning Objectives

Upon completion of this activity, participants will be able to:

- Identify the prevalence and epidemiology of nontuberculous mycobacteria (NTM) infection and traditional risk factors and presentation of pulmonary NTM infection.
- Construct an appropriate diagnostic strategy for patients with suspected NTM.

## MEDSCAPE CME Editor

**Beverly D. Merritt**, Technical Writer/Editor, *Emerging Infectious Diseases. Disclosure: Beverly D. Merritt has disclosed no relevant financial relationships.*

## MEDSCAPE CME AUTHOR

**Desiree Lie, MD, MSED**, Clinical Professor of Family Medicine, Director of Research and Faculty Development, University of California, Irvine at Orange, California. *Disclosures: Désirée Lie, MD, MSEd, has disclosed the following relevant financial relationship: served as a nonproduct speaker for: "Topics in Health" for Merck Speaker Services.*

## AUTHORS

*Disclosure:* ***Rachel M. Thomson, MD,*** *has disclosed no relevant financial relationships.k.*
