# Supplementary material for: Changing Epidemiology of Pulmonary Nontuberculous Mycobacteria Infections
Source: Emerg Infect Dis. 2010 Oct;16(10):1576–83. doi: 10.3201/eid1610.091201 (PMC3294381; doi:10.3201/eid1610.091201)
Supplement: Related texts

# Research article: Changing Epidemiology of Pulmonary Nontuberculous Mycobacteria Infections

**DOI:** 10.3201/eid1610.091201

**Authors:** Rachel M. Thomson, on behalf of the NTM working group at the Queensland TB Control Centre, Queensland Mycobacterial Reference Laboratory

**Affiliations:** Author affiliation: Queensland Tuberculosis Control Centre, Brisbane, Queensland, Australia

## Abstract

Nontuberculous mycobacteria (NTM) disease is a notifiable condition in Queensland, Australia. Mycobacterial isolates that require species identification are forwarded to the Queensland Mycobacterial Reference Laboratory, providing a central opportunity to capture statewide data on the epidemiology of NTM disease. We compared isolates obtained in 1999 and 2005 and used data from the Queensland notification scheme to report the clinical relevance of these isolates. The incidence of notified cases of clinically significant pulmonary disease rose from 2.2 (1999) to 3.2 (2005) per 100,000 population. The pattern of disease has changed from predominantly cavitary disease in middle-aged men who smoke to fibronodular disease in elderly women. *Mycobacterium intracellulare* is the main pathogen associated with the increase in isolates speciated in Queensland.

Worldwide, pulmonary disease caused by nontuberculous mycobacteria (NTM) appears to be increasing (*1*–*4*), yet accurate data to support this assumption are difficult to produce. Patients traditionally described are middle-aged men with underlying chronic lung disease, such as chronic obstructive pulmonary disease, who have upper lobe cavity formation and nodules of various sizes. An increasing number of patients have nodules, bronchiolitis, and bronchiectasis involving the middle lobe and lingula. These patients are more commonly female nonsmokers and have no preexisting lung disease (*5**,**6*).

NTM disease is not a reportable condition in most countries because no evidence of human-to-human transmission exists; therefore, it is not considered a public health concern. However, the organisms are ubiquitous in the environment, and substantial evidence shows that the environmental niche for *Mycobacterium intracellulare* (the most common pulmonary pathogen) is in biofilms lining suburban water pipes. Many NTM pathogens have been isolated from drinking water (*7*). Some clinicians believe the condition should be classified as an environmental health concern, similar to that caused by *Legionella* spp.

Globally, geographic variability in environmental exposure and prevalence of NTM disease is significant (*8*). Without detailed clinical information, differentiating between contamination of specimens, colonization/infection, and disease is difficult; laboratory reports of isolates do not always reflect the true incidence of disease. To determine if disease is present, sputum specimens and often a bronchoscopic sample of a patient’s lower respiratory tract must be collected. In addition, computed tomography scanning and clinical appointments with primary care providers and specialists are needed. Because this investigative process is costly to the healthcare system and the patient, accurate epidemiologic data on this condition should be of interest to public health experts.

Studies of avian versus human Mantoux testing in schoolchildren have shown that exposure to NTM organisms is common in Queensland (*9**–**11*). Therefore, since the introduction of TB control services, disease caused by NTM in Queensland has been notifiable. This practice has been continued primarily to avoid confounding of smear-positive cases with TB. The notification process provides a unique opportunity to study the clinical significance of isolates positive for NTM and the features such as age and sex, symptoms, underlying conditions, and radiology results of patients with disease, avoiding the inherent bias that occurs in case series that are reported by tertiary and quaternary referral centers. The incidence of clinically reported cases of pulmonary disease caused by *M. avium* complex (MAC) in Queensland has been increasing (1985, 0.63/100,000 population; 1994, 1.21/100,000; and 1999, 2.2/100,000). In 2005, the Queensland Tuberculosis Control Centre (QTBCC) revised the notification process to ensure collection of meaningful clinical data and follow-up of clinically significant NTM cases.

## Methods

At the time of this study, public hospitals requesting mycobacterial cultures forwarded all specimens to the Queensland Mycobacterial Reference Laboratory (QMRL). Requests in the private sector are processed by 2 main laboratories and a few smaller laboratories. The 2 private laboratories (representing >90% of the state’s private mycobacterial pathology service) identify mycobacteria in specimens and report as “atypical mycobacteria, not further specified.” After culture, not all isolates are forwarded to the QMRL for species identification. However, isolates are forwarded if the specimen is smear positive (to exclude TB), was obtained from a bronchial wash or a site other than sputum, and if >1 specimen is positive for that patient or the treating clinician requests it. In 1999, a specific research project was undertaken to capture all unspeciated isolates from private laboratories and better assess the true impact of mycobacteriology in the state. This study was approved by the Research Ethics Committee of the Princess Alexandra Hospital (138/98) in accordance with the Australian National Health and Medical Research Council guidelines. Because it proved quite labor intensive for all isolate reports to be forwarded for this study, it did not continue after 1999. Reports of all positive cultures identified by QMRL are forwarded to the QTBCC, which sends questionnaires to clinicians asking for the clinical significance of all mycobacterial isolates. Clinicians are provided with guidelines (as per American Thoracic Society/Infectious Disease Society of America [ATS/IDSA]) to assist in the diagnosis of significant disease.

More detailed clinical information is requested for patients who have disease, including details on predisposing conditions, radiologic and bacteriologic test results, and treatment received. Provision of this information is voluntary. Follow-up surveys evaluate any changes in regimen brought about by side effects or intolerance to drugs or failure of the starting regimen. Bacteriology is monitored for success of treatment. An update on the patient’s status is requested yearly for 3 years after treatment has stopped. All case files notified as clinically significant disease are reviewed by QTBCC clinicians to confirm concordance with ATS/IDSA criteria for disease (*12*).

Most nonpulmonary isolates grown by private laboratories are sent to the QMRL for speciation. For patients with nonpulmonary NTM isolates, the clinical significance of the isolate is sought by QTBCC; species of organism and site of infection is recorded. However, details on individual treatment regimens and outcomes are not collected.

Statistical calculations were performed by using OpenEpi: Open Source Epidemiologic Statistics for Public Health, version 2.3. (www.openepi.com). Population statistics were obtained from the Australian Bureau of Statistics (*13**,**14*), including population data for different age categories. The rate of positive NTM isolates is reported as number per 100,000 population, as are age-specific and gender-specific rates of pulmonary disease. Comparisons of statistics for 1999 and 2005 were made by using χ^2^ 2 × 2 tables in OpenEpi.

## Results

A more aggressive capture of single, positive, unspeciated isolates occurred in 1999; the total number of NTM isolates was 14.8/100,000 population. Because the number of speciated pulmonary isolates in each year (1999 and 2005) should not have been affected by the increase in data collection in 1999, the increase in speciated isolates for these 2 years most likely reflects the true increase in significant isolates (9.1–13.6/100,000 population). The total number of isolates in 2005 was 15.8/100,000 population. Assuming the proportion of unspeciated isolates remained constant, and on the basis of the increase in speciated isolates, we estimated the total number of isolates in 2005 to be 22.1/100,000 population.

The difference between the 2 years was due to the higher number of pulmonary isolates as demonstrated in Table 1. Speciated pulmonary isolates rose significantly, from 5.5 to 10.2/100,000 population.

**Table 1:** Comparison of numbers of nontuberculous mycobacteria isolates in 1999 and 2005, Queensland, Australia*

Isolates	No. isolates (rate/100,000 population; 95% CI)		p value
	1999	2005	
Total patient isolates	517† (14.8; 13.4–14.9)	631‡ (15.8; 14.3–16.9)	NS
Pulmonary	382 (10.9; 9.9–12.1)	488 (12.2; 11.1–13.3)	NS
Nonpulmonary	135 (3.9; 3.4–4.2)	143 (3.6; 3.1–4.0)	NS
Speciated isolates	318 (9.1; 8.2–10.2)	544 (13.6; 12.3–14.7)	<0.0000001
Pulmonary	192 (5.5; 4.5–6.6)	413 (10.2; 9.4–11.5)	<0.0000001
Nonpulmonary	119 (3.4; 3.0–3.8)	131 (3.3; 2.9–3.7)	NS

*CI, confidence interval; NS, not significant. Population of Queensland in 1999, 3.5 million; in 2005, 4 million. †In 1999, more smear-negative, single-positive unspeciated isolates were captured than usual, inflating these numbers. ‡The calculated number of positive isolates in 2005 was 22.1/100,000 population, based on the observed increase in speciated isolates, assuming the proportion of isolates not referred for speciation remained constant.

There were differences between the species of NTM isolated in 1999 and 2005 (Figure 1, Figure 2). The species accounting for most of the change in NTM isolates were *M. intracellulare* and *M. abscessus*. More of the MAC isolates are now identified by multiplex PCR as either *M. avium* or *M. intracellulare*. During 1999–2005, the number of *M. avium* isolates increased from 35 (1/100,000) to 62 (1.55/100000) and *M. intracellulare* from 77 (2.2/100,000) to 212 (5.3/100,000), while MAC decreased from 35 to 3. *M. abscessus* isolates increased from 23 (0.065/100, 000) in 1999 to 40 (1/100,000) in 2005. *M. fortuitum* isolates increased from 41(1.17/100,000) in 1999 to 50 (1.25/100,000) in 2005.

**Figure 1:** Changes in frequency of isolation for slow-growing (SG) nontuberculous mycobacteria (absolute numbers), Queensland, Australia, 1999 and 2005. MAC, *Mycobacterium avium* complex.

**Figure 2:** Changes in frequency of isolation for rapid-growing nontuberculous mycobacteria (absolute numbers), Queensland, Australia, 1999 and 2005.

The notified significance of pulmonary and extrapulmonary isolates is shown in Table 2. Of 488 patients with pulmonary NTM isolates, only 26.6% had significant disease overall. Percentages were slightly higher for patients with *M. intracellulare* isolates (39.4%), *M. avium* (33.3%), *M. kansasii* (52.6%), and much lower for patients infected with species traditionally thought more likely to be contaminants, such as *M. gordonae* (11.1%). Other rarer species were not thought to be associated with pulmonary disease (Table 3).

**Table 2:** Significance of nontuberculous mycobacteria isolates as notified, Queensland, Australia, 1999 and 2005*

Nontuberculous mycobacteria isolates	1999			2005	
	No. (%) isolates	Rate/100,000 population		No. (%) isolates	Rate/100,000 population
Pulmonary					
Significant	77 (20.2)	2.2		130 (26.6)	3.25
Under evaluation	1 (0.26)	0.03		52 (10.6)	1.3
Not significant	304 (79.6)	8.7		306 (62.7)	7.65
Extrapulmonary					
Significant	106 (78.5)	2.65		98 (68.5)	2.45
Under evaluation	0	–		4 (2.8)	–
Not significant	29 (21.5)	0.83		41 (28.7)	1.03

*–, not applicable.

**Table 3:** Clinical significance of nontuberculous mycobacteria isolates from pulmonary sites, Queensland, Australia, 2005*

Species	Significant	Under evaluation	Not significant	Total
Slow growers				
* Mycobacterium intracellulare*	79 (39.7)	22 (11.1)	98 (49.2)	199
* M. avium*	18 (33.3)	8 (14.8)	28 (48.3)	54
MAC	–	–	3 (100)	3
* M. kansasii*	10 (52.6)	3 (15.8)	6 (31.6)	19
* M. scrofulaceum*	3 (30)	–	7 (70)	10
* M. gordonae*	2 (11.1)	–	16 (88.9)	18
* M. lentiflavum*	–	–	5 (100)	5
* M. asiaticum*	–	–	3 (100)	3
* M. haemophilum*	–	–	1 (100)	1
* M. simiae*	–	–	2 (100)	2
Unspeciated	–	–	24 (100)	24
Rapid growers	–	–	–	–
* M. abscessus*	7 (24.1)	4 (13.8)	18 (62.1)	29
* M. chelonae*	3 (21.4)	2 (14.3)	9 (64.3)	14
* M. fortuitum*	1 (5.3)	1 (5.3)	17 (89.4)	19
* M. peregrinum*	–	1 (33.3)	2 (66.7)	3
* M. mucogenicum*	–	–	2 (100)	2
* M. nonchromogenicum*	–	1 (50)	1 (50)	2
Unspeciated	2 (28.6)	–	5 (71.4)	7
Atypical mycobacteria NFI	5(7.0)	10 (14.1)	56 (78.9)	71
Other	–	–	3 (100)	3
Total	130	52	306	488

*Values are given as no. isolates (% of total for species). MAC, *Mycobacterium avium* complex; –, not applicable; NFI, not further identified.

Of the isolates from nonpulmonary sites, the most common species was *M. fortuitum* (33/143), followed by *M. intracellulare* (12), *M. abscessus* (11), and *M. chelonae* (11). Overall, 68.5% of nonpulmonary isolates were felt to be associated with clinically significant NTM disease. Table 4 shows the statistical significance of these isolates according to species.

**Table 4:** Clinical significance of nontuberculous mycobacteria isolates from nonpulmonary sites, Queensland, Australia, 2005*

Species	Significant	Under evaluation	Not significant	Total
*Mycobacterium intracellulare*	7 (58.3)	–	5 (41.7)	12
*M. avium*	6 (66.7)	2 (22.2)	1 (11.1)	9
*M. scrofulaceum*	3 (60)	1 (20)	1 (20)	5
*M. gordonae*	1 (33.3)	–	2 (66.7)	3
*M. abscessus*	9 (81.8)	–	2 (18.2)	11
*M. chelonae*	8 (72.7)	–	3 (27.3)	11
*M. fortuitum*	23 (69.7)	3 (9.1)	7 (21.2)	33
*M. peregrinum*	7 (100)	–	–	7
*M. asiaticum*	–	–	1 (100)	1
*M. haemophilum*	1 (33.3)	–	2 (66.7)	3
*M. lentiflavum*	–	–	1 (100)	1
*M. mucogenicum*	–	–	1 (100)	1
*M. marinum*	–	–	1 (100)	1
*M. smegmatis*	1 (50)	–	1 (50)	2
*M. szulgai*	1 (100)	–	–	1
*M. ulcerans*	4 (100)	–	–	4
Atypical mycobacteria NFI	3 (27.3)	–	8 (72.7)	11
Slow grower unspeciated	1 (50)	–	1 (50)	2
Other	–	–	1 (100)	1
Total	97 (67.8)	6 (4.2)	40 (28)	143

*Values are given as no. isolates (% of total for species). –, not applicable; NFI, not further identified.

### Pulmonary Isolates

Of the 111 new notifications in 2005 with significant pulmonary disease that met ATS/IDSA criteria, *M. intracellulare* (63) was the most common pathogen, accounting for 56.8% of cases. *M. avium* (17) was the next most common (15.3%), followed by *M. kansasii* (9 [8.1%]). A total of 19 cases were repeat notifications (i.e., relapse/reinfection; *M. intracellulare* (15), *M. avium* (1), *M. kansasii* (1), *M. fortuitum* (1), and *M. abscessus* (1).

The age and sex distribution of patients with significant pulmonary disease differed in 1999 and 2005. In 1999, the absolute number of men with disease outnumbered women in all age groups with the exception of the >75-year age group. In 2005, women having NTM disease outnumbered men in all age groups >30 years; the difference was most marked in elderly persons. When adjusted for population within each age bracket and represented as rates per 100,000 (Figure 3), a significant increase was seen in women with disease who were 60–74 years of age (p = 0.0005), a decrease in men 30–44 years of age (p = 0.030) There was a combined overall increase in patients 60–74 years of age (p = 0.002) and a decrease in those 30–44 years of age (p = 0.013).

**Figure 3:** Age-adjusted rates for cases of nontuberculous mycobacteria disease among women (A), men (B), and all residents (C), Queensland, Australia, 1999 and 2005. *p = 0.0005; †p = 0.030; ‡p<0.005; §p = 0.057.

The radiologic pattern of disease has also changed (Figure 4). Most of the increase was in patients with noncavitary disease, from 25 (0.7/100,000) to 84 (2.1/100,000) cases (p<0.0001). A slight drop from 33 (0.9/100,000) cases of cavitary disease in 1999 to 28 (0.7/100,000) occurred in 2005 (p = 0.25).

**Figure 4:** Changes in the radiologic appearance of cases of nontuberculous mycobacteria disease, Queensland, Australia, 1999 and 2005.

## Discussion

In 2005, NTM were isolated from 15.6 persons per 100,000 population; however, the true number of isolates from that year is estimated at 22.1/100,000, a significant increase from 1999. Little change has been recorded in the isolation of NTM from nonpulmonary sites. However, the incidence of isolation of NTM from human pulmonary specimens has increased to 12.1/100,000 population. The incidence of notified significant pulmonary disease has increased from 2.2 to 3.2/100,000 population per year. This increase is largely due to an increase in the number of elderly women with noncavitary disease.

The observed changes in speciation are interesting epidemiologic observations, but their clinical relevance is yet to be defined. *M. intracellulare* is the main pathogen associated with the increase in disease, followed by *M. abscessus,* and *M. kansasii,* all clinically significant pulmonary pathogens. We have observed a change in the age and gender ratio. NTM disease has increased in elderly female patients who have predominantly nodular bronchiectasis, but disease has decreased in middle-aged men.

The epidemiology of NTM disease is difficult to study and equally difficult to report in a meaningful way. We have reported the incidence of new cases of clinically significant disease notified by clinicians that met the 1997 ATS/ISDA criteria (*12*) for disease. The true number of cases of disease may be an underestimate because the experience and attitudes of clinicians with regard to assessing the clinical significance of isolates is varied. It is likely that other cases of significant disease were not notified. The more recent ATS/IDSA standards of 2007 (*15*) have more relaxed microbiological criteria, and it is likely more case-patients would now be considered to have disease. Because NTM disease is a chronic disease, often over many years, the prevalence of disease will be much higher and more likely will reflect the impact on the healthcare system. Even with the current notification process in Queensland, we failed to capture all single, positive, unspeciated isolates.

A rising body of literature supports the observation by clinicians that the incidence and prevalence of disease is increasing. Other studies in Australia have also shown an increase over earlier time periods (*16**–**18*). The most recent report is from the Northern Territory. All isolates from 1989–1997 were examined (*16*). Disease incidence increased from 2.7 to 4.7 per 100,000 population between the first and second halves of this time period. Pang reported a 3-fold increase in *M. kansasii* disease in Western Australia during 1962–1982 and 1983–1987 (*19*). More recently Marras et al. (*3*) reported an increase in population prevalence of NTM isolates in Ontario, Canada from 2003 (9.1/100,000) to 2007 (14.1/100,000). Although adequate clinical data were not available for all patients, it is likely that the proportion of isolates responsible for disease increased in a similar manner. Skin testing by using MAC antigens to assess infection or at least exposure to NTM was initially used to demonstrate the considerable geographic distribution of NTM infection (*20**,**21*). More recently Khan et al. demonstrated an increase in skin test reactivity to *M. intracellulare* in 1971 and 1972 (11.2%) and 1999–2000 (16.6%) among representative US population cohorts (*22*).

If sensitization is a reflection of infection with NTM, then the observed increases cannot be attributed to the increased use of computed tomography scans, better laboratory techniques, or greater clinician awareness. Improved detection methods, such as the introduction of broth culture (MGIT BACTEC 960; Becton Dickinson, Franklin Lakes, NJ, USA) before the data collection of 1999, also cannot explain our observations. An increase in environmental exposure may have contributed to the increasing incidence of disease. The main environmental niche for *M. intracellulare* appears to be in biofilms lining drinking water pipes (*7*). *M. intracellulare* has been isolated from drinking water in Brisbane, as have *M. abscessus* and *M. kansasii* (R. Thomson, unpub. data). During the study period, Queensland had been affected considerably by drought, and restrictions to domestic water use had been enforced. The reduction in flow within distribution systems may have led to an increased chlorine degradation time and, hence, lower disinfectant levels at point of use. Previously, *M. scrofulaceum* was the main pathogen associated with childhood lymphadenitis. A change from chloramination to chlorination of water led to a reduction in the isolation of this species. The resurgence of this species in 2005 supports the notion of increased chlorine degradation, brought about by flow reductions but potentially also by temperature increases associated with climate change.

Changes in behavior associated with water restrictions may also have been a factor in resurgence of disease. The changes in turbulence that occur with the turning on and off of taps lead to disruptions in biofilms and release of mycobacteria into the water. However, some authors have suggested that patients may inhale aerosolized mycobacteria while showering (*23**,**24*). Recently, Feazel et al. (*25*) found mycobacterial DNA concentrated in the biofilms of showerheads in the United States. Queensland implemented a strong public awareness campaign promoting 4-minute showers to reduce water usage, which in theory should reduce exposure to mycobacteria through this route.

Several case series have reported gender imbalances in this disease. Earlier studies have demonstrated a male predominance with cavitary disease (*8**,**16**,**26*). More recent studies have shown a female predominance (*3**,**27**–**29*) of older patients with less preexisting lung disease. The differences between these groups of studies may be attributed to sampling bias and population differences. We have been able to demonstrate that, in fact, the pattern of disease is changing, from cavitary disease in middle-aged men who smoke to the nodular bronchiectatic form of disease in elderly women. Queensland government statistics suggest that the median age of the population is predicted to rise from 36.3 years in 2006 to 45 years in 2051, and the number of persons >65 years of age will increase 4-fold by 2051. As the vulnerable population increases, NTM disease is also likely to increase. Therefore, the impact of this disease on health budgets is worthy of further attention.

## References

[1] Henry MT, Inamdar L, O'Riordain D, Schweiger M, Watson JP. Nontuberculous mycobacteria in non-HIV patients: epidemiology, treatment and response. Eur Respir J. 2004;23:741–6. 10.1183/09031936.04.0011400415176690

[2] Marras TK, Daley CL. Epidemiology of human pulmonary infection with nontuberculous mycobacteria. Clin Chest Med. 2002;23:553–67. 10.1016/S0272-5231(02)00019-912370992

[3] Marras TK, Chedore P, Ying AM, Jamieson F. Isolation prevalence of pulmonary non-tuberculous mycobacteria in Ontario, 1997–2003. Thorax. 2007;62:661–6. 10.1136/thx.2006.07079717311842PMC2117272

[4] Thomson RM, Yew WW. When and how to treat pulmonary non-tuberculous mycobacterial diseases. Respirology. 2009;14:12–26. 10.1111/j.1440-1843.2008.01408.x19144045

[5] Levin DL. Radiology of pulmonary *Mycobacterium avium-intracellulare* complex. Clin Chest Med. 2002;23:603–12. 10.1016/S0272-5231(02)00009-612370996

[6] Kim RD, Greenberg DE, Ehrmantraut ME, Guide SV, Ding L, Shea Y, Pulmonary nontuberculous mycobacterial disease: prospective study of a distinct preexisting syndrome. Am J Respir Crit Care Med. 2008;178:1066–74. 10.1164/rccm.200805-686OC18703788PMC2720143

[7] Falkingham JO III, Norton CD, Le Chavallier MW. Factors influencing numbers of *Mycobacterium avium, Mycobacterium intracellulare,* and other mycobacteria in drinking water distribution systems. Appl Environ Microbiol. 2001;67:1225–31. 10.1128/AEM.67.3.1225-1231.200111229914PMC92717

[8] O'Brien RJ, Geiter LJ, Snider DE Jr. The epidemiology of nontuberculous mycobacterial diseases in the United States. Results from a national survey. Am Rev Respir Dis. 1987;135:1007–14.357900110.1164/arrd.1987.135.5.1007

[9] Abrahams EW, Silverstone H. Epidemiological evidence of the presence of non-tuberculous sensitivity to tuberculin in Queensland. Tubercle. 1961;42:487–99. 10.1016/S0041-3879(61)80138-413859022

[10] Abrahams EW, Harland RD. Sensitivity to avian and human PPD in Brisbane school children. Tubercle. 1967;48:79–94. 10.1016/S0041-3879(67)80002-36058036

[11] Abrahams EW, Harland RD. Studies with the purified protein derivative of human and avian tuberculins in South Queensland. Tubercle. 1968;49:192–209. 10.1016/0041-3879(68)90022-65690998

[12] American Thoracic Society. Diagnosis and treatment of disease caused by nontuberculous mycobacteria. Am J Respir Crit Care Med. 1997;156:S1–25.927928410.1164/ajrccm.156.2.atsstatement

[13] Australian Bureau of Statistics. Australian Demographic Statistics June Quarter 1999. Commonwealth of Australia; 1999 [cited 2010 Aug 12]. http://www.abs.gov.au/AUSSTATS/abs@.nsf/DetailsPage/3101.0Jun%201999?OpenDocument.

[14] Australian Bureau of Statistics. Australian Demographic Statistics December quarter 2005. Commonwealth of Australia; 2006 [cited 2010 Aug 12]. http://www.abs.gov.au/AUSSTATS/abs@.nsf/DetailsPage/3101.0Dec%202005?OpenDocument

[15] Griffith DE, Aksamit T, Brown-Elliott BA, Catanzaro A, Daley C, Gordin F, An official ATS/IDSA statement: diagnosis, treatment, and prevention of nontuberculous mycobacterial diseases. Am J Respir Crit Care Med. 2007;175:367–416. 10.1164/rccm.200604-571ST17277290

[16] O'Brien DP, Currie BJ, Krause VL. Nontuberculous mycobacterial disease in northern Australia: a case series and review of the literature. Clin Infect Dis. 2000; (4):958–67. 10.1086/31813611049777

[17] Edwards FG. Disease caused by ‘atypical’ (opportunistic) mycobacteria: a whole population review. Tubercle. 1970;51:285–95. 10.1016/0041-3879(70)90021-85495648

[18] Carruthers KJ, Edwards FG. Atypical mycobacteria in Western Australia. Am Rev Respir Dis. 1965;91:887–95.1429471210.1164/arrd.1965.91.6.887

[19] Pang SC. *Mycobacterium kansasii* infections in Western Australia. Respir Med. 1991;85:213–8. 10.1016/S0954-6111(06)80082-X1882111

[20] Palmer CE. Tuberculin sensitivity and contact with tuberculosis. Am Rev Tuberc. 1953;68:678–94.1310488210.1164/art.1953.68.5.678

[21] Edwards LB, Acquaviva FA, Livesay VT, Cross FW, Palmer CE. An atlas of sensitivity to tuberculin, PPD-B, and histoplasmin in the United States. Am Rev Respir Dis. 1969;99:1–132.5767603

[22] Khan K, Wang J, Marras TK. Nontuberculous mycobacterial sensitization in the United States: national trends over three decades. Am J Respir Crit Care Med. 2007;176:306–13. 10.1164/rccm.200702-201OC17507546

[23] Falkinham J. Environmental sources of *Mycobacterium avium* linked to routes of exposure. In: Pedley S BJ, Rees G, Dufour A, Cotruvo J, editors. Pathogenic mycobacteria in water. London: IWA Publishing; 2004. p. 26–38.

[24] Marras TK, Wallace RJ Jr, Koth LL, Stulbarg MS, Cowl CT, Daley CL. Hypersensitivity pneumonitis reaction to *Mycobacterium avium* in household water. Chest. 2005;127:664–71. 10.1378/chest.127.2.66415706013

[25] Feazel LM, Baumgartner LK, Peterson KL, Frank DN, Harris JK, Pace NR. Opportunistic pathogens enriched in showerhead biofilms. Proc Natl Acad Sci U S A. 2009;106:16393–9. 10.1073/pnas.090844610619805310PMC2752528

[26] Ahn CH, Lowell JR, Onstad GD, Shuford EH, Hurst GA. A demographic study of disease due to *Mycobacterium kansasii* or *M. intracellulare-avium* in Texas. Chest. 1979;75:120–5. 10.1378/chest.75.2.120421546

[27] Freeman J, Morris A, Blackmore T, Hammer D, Munroe S, McKnight L. Incidence of nontuberculous mycobacterial disease in New Zealand, 2004. N Z Med J. 2007;120:50–6.17589548

[28] Prince DS, Peterson DD, Steiner RM, Gottlieb JE, Scott R, Israel HL, Infection with *Mycobacterium avium* complex in patients without predisposing conditions. N Engl J Med. 1989;321:863. 10.1056/NEJM1989092832113042770822

[29] Huang JH, Kao PN, Adi V, Ruoss SJ. *Mycobacterium avium-intracellulare* pulmonary infection in HIV-negative patients without preexisting lung disease: diagnostic and management limitations. Chest. 1999;115:1033–40. 10.1378/chest.115.4.103310208205

# Research article: Changing Epidemiology of Pulmonary Nontuberculous Mycobacteria Infections

**DOI:** 10.3201/eid1610.091201

**Authors:** Rachel M. Thomson, on behalf of the NTM working group at the Queensland TB Control Centre, Queensland Mycobacterial Reference Laboratory

**Affiliations:** Author affiliation: Queensland Tuberculosis Control Centre, Brisbane, Queensland, Australia

## Earning CME Credit

To obtain credit, you should first read the journal article. After reading the article, you should be able to answer the following, related, multiple-choice questions. To complete the questions and earn continuing medical education (CME) credit, please go to **www.medscapecme.com/journal/eid**. Credit cannot be obtained for tests completed on paper, although you may use the worksheet below to keep a record of your answers. You must be a registered user on Medscape.com. If you are not registered on Medscape.com, please click on the New Users: Free Registration link on the left hand side of the website to register. Only one answer is correct for each question. Once you successfully answer all post-test questions you will be able to view and/or print your certificate. For questions regarding the content of this activity, contact the accredited provider, CME@medscape.net. For technical assistance, contact CME@webmd.net. American Medical Association’s Physician’s Recognition Award (AMA PRA) credits are accepted in the US as evidence of participation in CME activities. For further information on this award, please refer to http://www.ama-assn.org/ama/pub/category/2922.html. The AMA has determined that physicians not licensed in the US who participate in this CME activity are eligible for *AMA PRA Category 1 Credits*™. Through agreements that the AMA has made with agencies in some countries, AMA PRA credit is acceptable as evidence of participation in CME activities. If you are not licensed in the US and want to obtain an AMA PRA CME credit, please complete the questions online, print the certificate and present it to your national medical association.

## Article Title: Changing Epidemiology of Pulmonary Nontuberculous Mycobacteria Infections

## CME Questions

**1. Which of the following patients is at most risk for pulmonary nontuberculous mycobacteria (NTM) infection?**A. A middle-aged female smoker with immune suppression with middle lobe infectionB. A middle-aged man with chronic obstructive pulmonary disease with upper lobe infectionC. An elderly woman with chronic obstructive pulmonary disease with diffuse nodules throughout the lungD. An elderly man with lung fibrosis and immune suppression with bilateral lung disease**2. Which of the following diagnostic strategies is most appropriate for patients with suspect pulmonary NTM infection?**A. Multiple early-morning gastric aspiratesB. Lung biopsyC. Multiple sputa and bronchoscopic samplingD. Chest x-ray

## Activity Evaluation

**Table:** 

1. The activity supported the learning objectives.				
Strongly Disagree				Strongly Agree
1	2	3	4	5
2. The material was organized clearly for learning to occur.				
Strongly Disagree				Strongly Agree
1	2	3	4	5
3. The content learned from this activity will impact my practice.				
Strongly Disagree				Strongly Agree
1	2	3	4	5
4. The activity was presented objectively and free of commercial bias.				
Strongly Disagree				Strongly Agree
1	2	3	4	5
